# Effects of High-Inorganic-Phosphorus Diet on Intestinal Mucosal Injury and Immune Alteration in Mice

**DOI:** 10.3390/nu18101590

**Published:** 2026-05-16

**Authors:** Zongchao Sun, Shiya Huang, Yuxin Zhao, Yunhan Luan, Yinuo Wang, Runzhe Wang, Weiwei Wu, Danli Huang, Jiankang Liu, Yinghui Zhang

**Affiliations:** 1 School of Life Sciences and Health, University of Health and Rehabilitation Sciences, Qingdao 266113, China; sunzongchao@uhrs.edu.cn (Z.S.); huangshiya@uhrs.edu.cn (S.H.); luanyunhan1@uhrs.edu.cn (Y.L.); wangyinuo1@uhrs.edu.cn (Y.W.); wangrunzhe1@uhrs.edu.cn (R.W.); jkliu@uhrs.edu.cn (J.L.); 2 School of Food Science and Engineering, Foshan University, Foshan 528225, China; zhaoyx202407@163.com (Y.Z.); 19849501850@163.com (W.W.); 13097746601@163.com (D.H.)

**Keywords:** inorganic phosphate, intestinal mucosa, immune alterations, IgE

## Abstract

**Background/Objectives**: Excessive dietary inorganic phosphate (Pi) as a food additive poses potential health risks. **Methods**: This study investigated the impact of excessive dietary inorganic phosphate on intestinal and immune homeostasis in mice using gradient Pi exposure combined with an inflammatory model. **Results**: Pi overload induced atrophy in the thymus, spleen, and kidney; damaged the intestinal barrier; reduced the villus height-to-crypt-depth ratio; and decreased goblet cell numbers. Altered levels of serum sIgA and IgE, as well as intestinal IgA, IgG, IgE, and IgM, together with decreased IFN-α, indicated altered levels of immunoglobulins and cytokines under Pi treatment. Proteomic analysis revealed differential expression of key proteins, including CNTFR and Bcl2l1 in the JAK/STAT pathway and metabolic regulators CPT1α and IDH1, when comparing Pi-treated mice with the control group. **Conclusions**: These preliminary findings suggest that Pi may affect intestinal mucosal barrier function and systemic immune response through immune regulation and mitochondrial metabolic pathways, providing preliminary insight into the potential health implications of Pi overconsumption in humans.

## 1. Introduction

Phosphorus is an essential nutrient for the human body, playing a critical role in key physiological processes such as energy metabolism, bone tissue formation, the maintenance of cell membrane structure, and intracellular signal transduction [1,2,3]. Dietary phosphorus is mainly divided into two categories: organic and inorganic. In natural foods, phosphorus primarily exists in organic forms, with an absorption rate of approximately 10%~30%. In contrast, inorganic phosphates, widely used as food additives, have a bioavailability of nearly 100% [4,5]. Inorganic phosphates are extensively applied in the food industry, pharmaceuticals, and animal feed. According to statistics, over 70% of frozen and dried foods, more than 65% of pre-packaged meat products, over 55% of baked goods, and more than 50% of soups and yogurts in supermarkets contain inorganic phosphorus additives [6,7]. It is estimated that thousands of food products containing phosphorus additives are available for human consumption.

Currently, the global average per capita phosphorus intake is nearly twice the recommended nutritional intake (RNI), with a trend toward further increase. In China, the RNI for phosphorus is 720 mg/day for adults, while the tolerable upper intake level (UL) is 3500 mg/day; in the United States, the RNI is 700 mg/day and the UL is 4000 mg/day; and in the European Union, the RNI is 550 mg/day and the UL is 2500 mg/day. Excessive intake of inorganic phosphorus, particularly from food additives, has become a widespread public health problem worldwide, especially in countries and regions with a high consumption of ultra-processed foods. Notably, inorganic phosphorus additives contribute 60–80% of total phosphorus intake in processed meats and over 90% in carbonated beverages. This means that even at the same total phosphorus intake, individuals consuming large amounts of processed foods are exposed to significantly higher levels of bioavailable inorganic phosphate than those primarily consuming natural foods, which may lead to different health outcomes. The lack of mandatory labeling of inorganic phosphorus on food labels globally is a key factor contributing to its uncontrolled intake among the public [8].

Excessive intake of inorganic phosphorus poses multiple health risks, such as adverse mental health symptoms, disruption of calcium–phosphorus balance, and impairments in bone and liver function, and is associated with coronary and heart valve calcification, worsening renal function, reduced exercise tolerance, and increased all-cause mortality [9,10,11]. The pathogenesis of high phosphorus intake promotes cardiac valve calcification by inducing osteogenic differentiation of a subset of valvular interstitial cells [12]. Calcium–phosphorus balance in the human body is regulated by the FGF23/α-klotho signaling pathway. Higher phosphate intake raises FGF23, without a parallel increase in α-klotho, and thus increases the FGF23/α-klotho ratio. An increased serum FGF23 concentration or FGF23/α-klotho ratio was positively linearly related to diabetes and cardiovascular diseases [13,14].

The intestine, as the primary site of digestion and absorption, is also an important immune organ in the human body. However, the systemic immune effects of excessive inorganic phosphate intake, particularly the combined effects with mild inflammatory stimulation, remain poorly understood. This study employed a mouse inflammation model stimulated with a low dose of LPS and investigated the impact of a high-inorganic-phosphorus diet on the intestinal immune barrier and systemic immune function. The investigative methods included organ indices, intestinal morphology, ELISA of immunoglobulin expression, and proteomic changes. The findings aim to provide a preliminary theoretical basis for future research on the scientific regulation of dietary phosphorus intake and offer a hypothesis that the occurrence of food hypersensitivity reactions is related to the excessive intake of inorganic phosphorus.

## 2. Materials and Methods

### 2.1. Materials

A total of 40 SPF-grade female BALB/c mice were selected as experimental subjects. The mice were supplied by the Guangdong Provincial Medical Laboratory Animal Center (Experimental Animal License No.: Yue Shi Zheng (2019) 05073); they weighed 20 ± 2 g and were 6–8 weeks old. This study employed LPS (lipopolysaccharide) (Sigma, Kawasaki, Japan), trisodium phosphate food additive (Zhengzhou Gaoyan Biotechnology Co., Ltd., Zhengzhou, China), Carnoy’s fixative, HE staining kit, AB-PAS staining kit (Servicebio, Wuhan, China), IL-1β ELISA kit (Yikexai Biotechnology Co., Ltd., Suzhou, China), IFN-α ELISA kit, IgG ELISA kit (Huamei Bio, Wuhan, China), sIgA ELISA kit, IgA ELISA kit, IgE ELISA kit (Shanghai Sangon, Shanghai, China), proteinase inhibitor (Thermo Fisher Scientific, Waltham, MA, USA, A32953), sodium deoxycholate (Sigma-Aldrich, St. Louis, MO, USA, 30970), chloroacetamide (Sigma-Aldrich, 22790), and tri(2-carboxyethyl)phosphine (Sigma-Aldrich, C4706). The equipment used was a microplate reader (Thermo Fisher Scientific, Waltham, MA, USA), an Omni Bead Ruptor Elite 24 bead homogenizer (OMNI, Dallas, TX, USA), nanoElute liquid chromatography (Bruker Corporation, Billerica, MA, USA), and a timsTOF Pro mass spectrometer (Bruker Corporation, Billerica, MA, USA).

#### 2.1.1. Animal Experiments

The three trisodium phosphate dose gradients (100 mg/kg, 200 mg/kg, 400 mg/kg) used in this study were designed based on allometric scaling by body surface area (the FDA/EFSA-recommended gold standard for animal-to-human dose translation), which fully accounts for interspecies differences in metabolism, absorption kinetics, and exposure routes. The doses are intended to simulate excessive dietary phosphate exposure in humans rather than normal physiological intake: 100 mg/kg trisodium phosphate corresponds to the average daily phosphorus intake of adult males in the United States (1665 mg/day); 200 mg/kg corresponds to high intake in people who consume large amounts of processed foods for a long time (2610 mg/day); and 400 mg/kg corresponds to an extreme exposure level slightly above the global unified tolerable upper intake level (4000 mg/day), which is used to assess the potential safety risk boundary. It is important to note that this dose translation is approximate and does not account for all interspecies differences in phosphate homeostasis. Human responses to phosphate exposure may differ significantly from those observed in mice.

During the animal experiments, mice were labeled using a staining method. Forty mice were randomly assigned to eight groups using a random number generator: blank control group (Control), LPS model group (LPS), low-dose phosphate group (LIP), medium-dose phosphate group (MIP), high-dose phosphate group (HIP), low-dose phosphate–LPS group (LIP–LPS), medium-dose phosphate–LPS group (MIP–LPS), and high-dose phosphate–LPS group (HIP–LPS). The Control and LPS model groups received daily oral administration of 0.9% sodium chloride solution at 0.2 mL/10 g body weight. The low-dose trisodium phosphate and low-dose trisodium phosphate–LPS groups received daily oral administration of trisodium phosphate solution at 100 mg/kg body weight via 0.2 mL/10 g body weight. The medium-dose trisodium phosphate group and medium-dose trisodium phosphate–LPS group received 0.2 mL/10 g body weight of 200 mg/kg trisodium phosphate solution daily; the high-dose trisodium phosphate group and high-dose trisodium phosphate–LPS group received 0.2 mL/10 g body weight of 400 mg/kg trisodium phosphate solution daily. Gavage was administered once daily for 15 consecutive days. On day 15, the mice received their final gavage. Three hours post-gavage, LPS solution (0.05 mg/mL) was intraperitoneally injected in the LPS, LIP–LPS, MIP–LPS, and HIP–LPS groups, respectively. During the experiment, the mice were monitored for behavioral changes. Mouse body weights were weighed and recorded daily. Twenty-four hours after the final gavage, the experimental mice were euthanized by cervical dislocation. Their small intestines, livers, thymuses, and spleens were collected for subsequent experimental studies. This study was designed as an exploratory investigation to identify potential effects of a high-inorganic-phosphorus diet on intestinal and immune homeostasis. The sample size of 5 mice per group is standard for preliminary exploratory studies in this field and is sufficient to detect large effect sizes (Cohen’s d > 0.8), which were observed for all key findings in this study. However, this sample size is underpowered for detecting small to moderate effects and for definitive confirmation of causal relationships. All conclusions drawn from this study should be considered preliminary and require validation in larger-scale studies.

#### 2.1.2. Preparation of Paraffin Sections

The detailed experimental procedures for the preparation of paraffin sections are described in the Supplementary Materials.

#### 2.1.3. Preparation of Intestinal HE-Stained Sections

The detailed experimental procedures for the preparation of HE-stained sections are described in the Supplementary Materials.

All histomorphometric measurements were performed in a blinded manner by two independent researchers. Standardized jejunal segments (10 cm distal to the ligament of Treitz) were used for all samples, and 5–6 villi/crypts were measured per tissue section to minimize sampling variation. A total of 3–5 randomly selected mouse intestinal villi were photographed to measure villus length and crypt depth, and the ratio of villus length to crypt depth was calculated.

#### 2.1.4. Preparation of AB-PAS-Stained Intestinal Sections

The detailed experimental procedures for the preparation of AB-PAS-stained intestinal sections are described in the Supplementary Materials.

All goblet cell counting was performed in a blinded manner by two independent researchers using the same standardized jejunal segments as above. After preparation, the morphological changes in small intestinal tissues were observed under an inverted light microscope, with the intestinal lumen villi photographed, and goblet cells on the villi counted and analyzed.

#### 2.1.5. ELISA Assay for Detecting Serum sIgA and IgE Levels in Mice

The detailed experimental procedures for the ELISA are described in the Supplementary Materials.

All ELISAs were performed in technical triplicates to ensure result reliability. The optical density (OD) values of the standard solutions and samples were measured at 450 nm using the pre-warmed microplate reader, and the actual concentrations of sIgA and IgE in each serum sample were calculated based on the standard curve generated from the standard solution assay results.

#### 2.1.6. ELISA for Detecting IFN-α and IL-1β Levels, as Well as IgE, IgG, IgM, and IgA Expression in Mouse Small Intestinal Tissue

The detailed experimental procedures for the ELISA are described in the Supplementary Materials.

All ELISAs were performed in technical triplicates to ensure result reliability. The optical density (OD) values of the standards and test samples were measured at a wavelength of 450 nm using a pre-warmed microplate reader, and the actual concentrations of interferon-α (IFN-α), interleukin-1β (IL-1β), immunoglobulin E (IgE), immunoglobulin G (IgG), immunoglobulin M (IgM), and immunoglobulin A (IgA) in each small intestinal tissue sample were calculated based on the standard curve constructed from the standard OD values.

#### 2.1.7. Immunofluorescence Detection of IgA Cell Expression in the Intestinal Tract of Experimental Mice

The detailed experimental procedures for immunofluorescence detection are described in the Supplementary Materials.

All immunofluorescence quantification was performed in a blinded manner by two independent researchers. Images were captured at the excitation wavelengths of 330–380 nm (for DAPI), 488 nm, 465–495 nm (for CY3), and 515–555 nm (for CY5), as well as their corresponding emission wavelengths: 510–560 nm, 590 nm, 608–648 nm, and 672–712 nm.

#### 2.1.8. Proteomics Sample Preparation

Approximately 20 mg of mouse spleen tissue was weighed and mixed with phosphate-buffered saline (PBS) containing protease inhibitors, followed by thorough vortexing. Lysis buffer was then added, and the mixture was incubated in a 95 °C water bath for 5 min. The sample was transferred to a grinding tube and homogenized using a bead mill homogenizer (Thermo Fisher Scientific, Waltham, MA, USA, A32953) under the following conditions: speed 7.1 m/s, with cycles of 20 s on and 20 s off, repeated for five cycles. After homogenization, the sample was transferred to a new centrifuge tube, and contact ultrasonication was performed on ice for protein extraction at 30% energy (4 s on, 8 s off) for a total duration of 3 min. The extracted protein was centrifuged at 16,000× *g* for 5 min, and the supernatant was collected for the determination of protein concentration via the bicinchoninic acid (BCA) assay. Next, 100 μg of protein was diluted and mixed with Tris-HCl buffer (pH 8.8). Trypsin was added at a protein-to-enzyme ratio of 25:1; the mixture was vortexed thoroughly and incubated at 37 °C on a shaking mixer (1000 rpm) for 16 h. The pH of the mixture was adjusted to 3 using 10% formic acid (FA), and the sample was centrifuged at 16,000× *g* for 5 min. The resulting supernatant was collected, desalted using an automated desalting system, and dried at 45 °C. The dried sample was resuspended in 30 μL of 0.1% FA, the peptide concentration was measured, and the sample was prepared for instrumental analysis.

#### 2.1.9. LC-MS/MS Analysis

Cohort samples were analyzed using a Bruker nanoElute liquid chromatography system coupled to a Bruker timsTOF Pro mass spectrometer. A 30 cm × 75 μm column packed with 1.9 μm C18 particles (120 Å, Dr. Maisch GmbH, Ammerbuch, Germany) was employed. The nanoElute gradient was set as follows: 2–4% mobile phase B for the first 5 min, 4–22% B for 5–70 min, 22–35% B for 70–90 min, 35–80% B for 90–100 min, and 80% B for 100–110 min. Mobile phase composition: phase A consisted of water and 0.1% formic acid solution; phase B consisted of acetonitrile and 0.1% formic acid solution. The overall flow rate was 300 nL/min but increased to 500 nL/min during the 5 min preceding formal detection. Bruker timsTOF Pro mass spectrometer parameters: ion source voltage set to 4.5 kV, ion source temperature set to 180 °C, and ion source flow rate of 3 L/min. Data acquisition mode employed DIA-PASEF. The primary mass spectrum m/z range was 300–1500, with ion drift set to 1/K0. The scanning range was 0.75–1.40 V s/cm^2^, and the ramp-up time was set to 100 ms. For secondary spectrum acquisition, the peptide m/z range was 400–1200, the charge state was set to 0–5, the mass isolation window was set to 25 Th, the DIA-PASEF window count was set to 64, and the total method cycle time was set to 1.17 s. To prevent duplicate peptide scanning, the dynamic exclusion time (release after) for tandem mass spectrometry was set to 30 s.

#### 2.1.10. Statistical Analysis

Data obtained from this experiment were analyzed using GraphPad Prism 11.0 and R 4.3.1 software. All statistical tests were two-sided with the significance level set at α = 0.05.

For all intergroup comparisons in the 2 × 4 factorial design (phosphate treatment × LPS stimulation), two-way ANOVA was used to evaluate the independent effects of inorganic phosphate treatment, LPS stimulation, and their interaction. The results of the main effects and interaction effects are presented in Supplementary Table S1. For all significant interactions (*p* < 0.05), Tukey’s HSD post hoc test was performed for pairwise comparisons to correct for Type I errors. All reported *p*-values in the figures and text are adjusted values from these post hoc tests.

For pairwise comparisons between two groups, an independent-samples t-test was used.

Prior to statistical analysis, all continuous variables were tested for normality using the Shapiro–Wilk test and for homogeneity of variances using Levene’s test. Data meeting both assumptions are presented as the mean ± standard deviation (SD). Non-normally distributed data were rank-transformed before analysis, or analyzed using nonparametric tests (Mann–Whitney U test for two-group comparisons, and Kruskal–Wallis test followed by Dunn’s post hoc test for multiple-group comparisons).

Effect sizes were calculated and reported: partial eta-squared (η^2^) for main effects and interaction effects in two-way ANOVA, and Cohen’s d for all significant pairwise comparisons. All key findings showed large effect sizes (partial η^2^ > 0.14, Cohen’s *d* > 0.8).

Pearson correlation analyses were performed to quantify the linear relationships between inorganic phosphate dose and primary outcome measures separately in the non-LPS and LPS-stimulated groups.

For proteomic analysis, the Benjamini–Hochberg false discovery rate (FDR) correction was applied to adjust *p*-values for multiple comparisons. Only proteins with adjusted *p*-values < 0.05 and fold changes >1.5 were considered significantly differentially expressed.

Protein–protein interaction network analysis was visualized using the String website. Data analysis and quality control were performed using Python 3.8 algorithms.

## 3. Results

### 3.1. A Diet High in Inorganic Phosphate Impairs the Organ Indices and the Integrity of the Intestinal Mucosal Barrier in Mice

**Figure 1:**
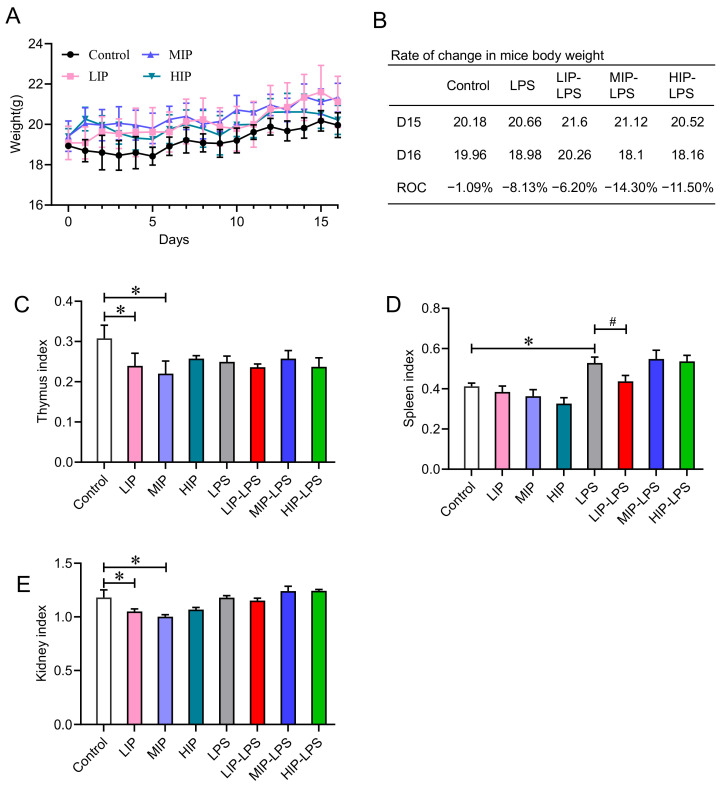
Effects of a high-inorganic-phosphorus diet on body weight and organ indices in mice. (**A**) Trend chart of body weight changes in mice receiving low, medium, and high doses of phosphate via gastric lavage for 15 days, *n* = 5. (**B**) Rate of change in body weight of mice in the inflammation model group, *n* = 5. (**C**) Effect of high-inorganic-phosphorus consumption on thymus index in mice, *n* = 5. (**D**) Effect of high-inorganic-phosphorus consumption on spleen index in mice, *n* = 5. (**E**) Effect of high-inorganic-phosphorus consumption on kidney index in mice, *n* = 5. All data are shown as the mean ± SEM. * *p* < 0.05 vs. controls, ^#^ *p* < 0.05 vs. LPS, determined by two-way ANOVA coupled to Tukey’s HSD post hoc test.

The intestine is the primary site for digestion and absorption, and it also represents the largest and most complex immune organ in the human body, undertaking critical functions. The intestinal mucosa acts as a crucial barrier separating the internal milieu from the luminal environment. When its integrity is compromised, this may permit the invasion of exogenous harmful substances, triggering inflammation and tissue damage. This study employed a 15-day intragastric administration of varying doses of inorganic phosphate (100, 200, 400 mg/kg) to mice, followed by the intraperitoneal injection of LPS to establish an acute inflammation model, aiming to investigate the impact of the food additive inorganic phosphate on the immune system and organ function under inflammatory challenge. Body weight monitoring during the experiment revealed an increase in weight across all phosphate-dosed groups compared to the pre-administration levels, whereas no significant change was observed in the blank control group (Figure 1A). However, upon LPS-induced inflammatory challenge, all experimental groups exhibited a significant decrease in body weight (Figure 1B).

Analysis of organ indices indicated that inorganic phosphate intake led to a decrease in the thymus index and spleen index, suggesting that phosphate overload may induce potential morphological and weight changes in immune and metabolic organs (Figure 1C,D). The kidney index also showed a dose-dependent decrease, indicating adverse effects of high phosphate intake on renal health (Figure 1E). Notably, under the acute inflammatory state induced by LPS, the spleen and kidney indices showed a recovery, providing further macroscopic evidence supporting the inference that inorganic phosphate may exert immunomodulatory effects (Figure 1C–E). These preliminary findings suggest that excessive inorganic phosphorus intake may be associated with reduced indices of immune organs such as the thymus and spleen and structural alterations of intestinal villi in mice.

**Figure 2:**
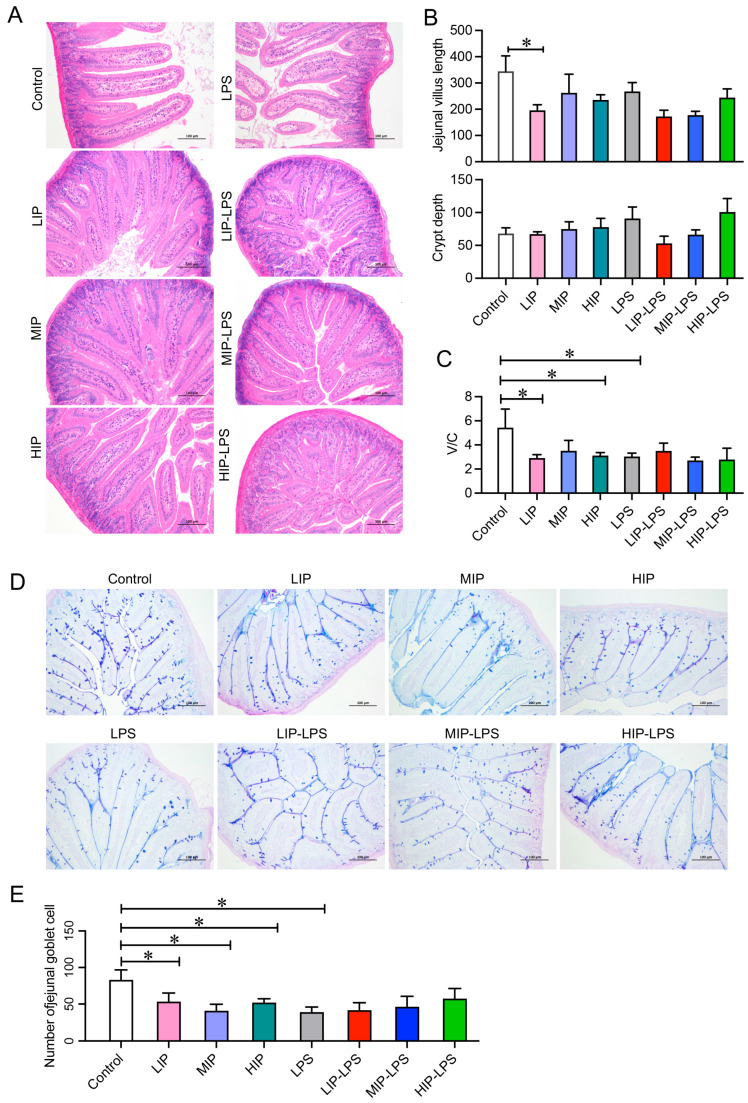
A high-inorganic-phosphorus diet induces intestinal injury and impairs the repair of the mucosal barrier in mice. (**A**) Pathological observation of HE-stained sections of mouse small intestine tissue (100×), *n* = 5. (**B**) Histogram of jejunal villus length and crypt depth in mice, *n* = 5. (**C**) Histogram of the ratio of villi length to crypt depth in the small intestine of mice, *n* = 5. (**D**) AB-PAS-stained sections of mouse small intestine tissue were observed (100×), *n* = 5. (**E**) Histogram of mouse jejunal goblet cell count, *n* = 5. All data are shown as the mean ± SEM. * *p* < 0.05 vs. controls, determined by two-way ANOVA coupled to Tukey’s HSD post hoc test.

The effects of a high-inorganic-phosphate diet on intestinal barrier structure were systematically evaluated further via histomorphological analysis. H&E staining results showed that compared to the blank control group, all phosphate-intervention groups exhibited morphological alterations including shortened average intestinal villi height and increased crypt depth, with the 100 mg/kg phosphate treatment group showing a significant reduction of approximately 50% in villus length (Figure 2A–C). The ratio of villus height to crypt depth (V/C ratio) was significantly decreased in all treatment groups, indicating villi atrophy and crypt hyperplasia (Figure 2C). AB-PAS staining and cell counting analysis demonstrated that the number of goblet cells in the phosphate-intervention groups was significantly lower than in the control group (Figure 2D,E). These results collectively indicate that inorganic phosphate intake alters intestinal structure and immune parameters in mice by inducing villus atrophy and crypt hyperplasia and reducing the number of goblet cells.

In summary, excessive intake of inorganic phosphate as a food additive not only exerts negative effects on organs such as the thymus, spleen, and kidneys, but also impairs the structure and function of the intestinal mucosal barrier, highlighting its potential risks in terms of immunomodulation and intestinal health.

### 3.2. Effects of a High Inorganic Phosphate Diet on the Intestinal Immune Environment in Mice

**Figure 3:**
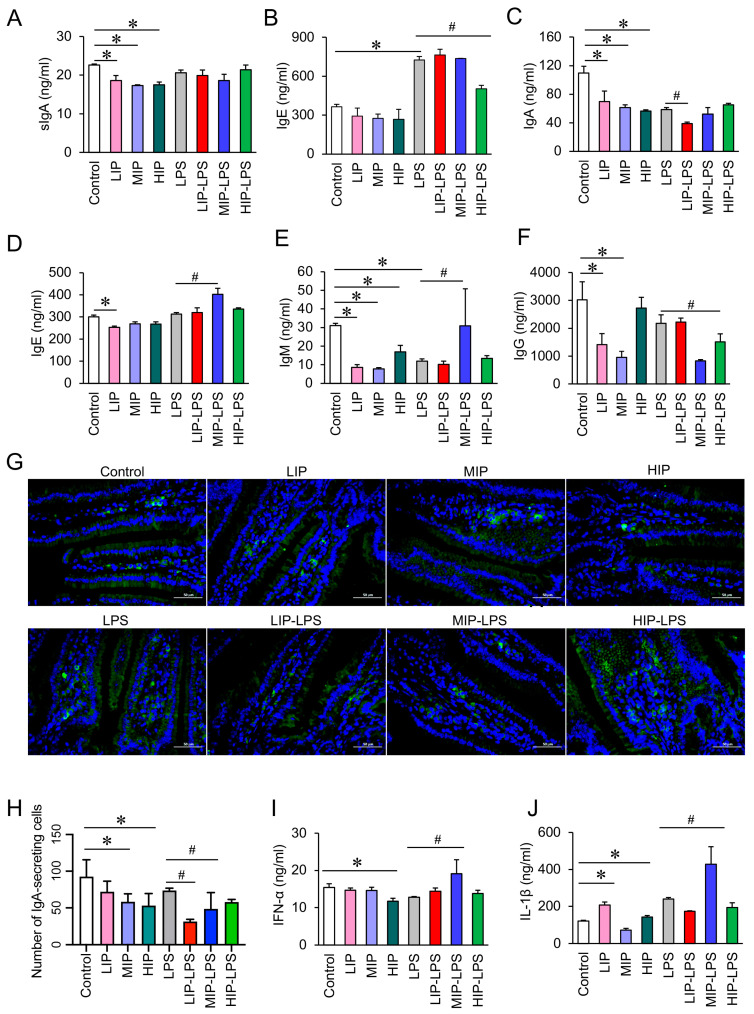
Inorganic phosphates impair the intestinal immune environment in mice by affecting immunoglobulins and pro-inflammatory cytokines. (**A**) Expression of sIgA in mouse serum, *n* = 5. (**B**) Expression of IgE in mouse serum, *n* = 5. (**C**) IgA content in the mouse small intestine, *n* = 5. (**D**) IgE content in the mouse small intestine, *n* = 5. (**E**) IgM content in the mouse small intestine, *n* = 5. (**F**) IgG content in the mouse small intestine, *n* = 5. (**G**) Immunofluorescence was used to observe the secretion of IgA cells in the intestinal lumen of mice (200×), *n* = 5. (**H**) Histogram of the number of IgA-secreting cells in the jejunal mucosa of mice (immunofluorescence, 200×), *n* = 5. (**I**) Expression of IFN-α in mouse small intestinal tissue, *n* = 5. (**J**) Expression of Il-1β in mouse small intestinal tissue, *n* = 5. All data are shown as the mean ± SEM. * *p* < 0.05 vs. controls, ^#^ *p* < 0.05 vs. LPS, determined by two-way ANOVA coupled to Tukey’s HSD post hoc test.

There is a significant correlation between damage to the intestinal mucosal barrier and food allergies. On the basis of detecting the structural damage caused by sodium phosphate to the intestinal mucosa, we measured the protein expression levels of immunoglobulins (IgA, sIgA, IgE, IgM, IgG) and pro-inflammatory cytokines (IL-1β, IFN-α) in the blood and small intestine of mice. The results showed that gavage of sodium phosphate significantly reduced the levels of sIgA in mouse serum (Figure 3A) and IgA in the small intestine (Figure 3C), and the number of IgA secreting cells in the small intestine wall also decreased (Figure 3G,H). Gavage of sodium phosphate also significantly reduced the protein expression of IgE (Figure 3D), IgM (Figure 3E), and IgG (Figure 3F) in the small intestine of mice.

Although gavage of sodium phosphate alone did not significantly alter the expression of IgE in mouse serum (Figure 3B), induction with adjuvant LPS led to a significant increase in the expression of IgE (Figure 3D), IgM content (Figure 3E), IFN-α (Figure 3I), and IL-1β (Figure 3J) in the small intestine tissue of mice administered 200 mg/kg sodium phosphate (MIP–LPS group).

In addition, gavage of 200 mg/kg sodium phosphate significantly inhibited the LPS-induced IgG levels (Figure 3F), reflecting a decrease in humoral immune function and possible impairment of the body’s ability to resist pathogens.

In summary, inorganic phosphate alters the expression profile of immunoglobulins and pro-inflammatory cytokines in the mouse intestine. This study provides evidence that inorganic phosphate may induce intestinal immune alterations and modulate inflammatory responses, suggesting that long-term or excessive intake of inorganic phosphate food additives may pose potential risks to immune function.

### 3.3. Proteomic Analysis of the Effects of a High-Phosphate Diet on the Immune System of Mice

Based on data-independent acquisition (DIA) quantitative proteomics technology, this study systematically analyzed the impact of a high-phosphate diet on the protein expression profile in the mouse spleen. Quality control of the data analysis showed low coefficients of variation and high consistency among samples in each group (Supplementary Figure S1A), with significant correlations in protein quantification between samples (Supplementary Figure S1B), indicating reliable data quality.

Through differential expression analysis (screening criteria: adjusted *p* < 0.05 and fold change >1.5), a number of significantly differentially expressed proteins (DEPs) were identified across different comparison groups. Specifically, 187, 132, and 77 DEPs were identified when comparing the blank control group with the low-, medium-, and high-dose phosphate groups, respectively. Conversely, comparisons between the LPS model group and the corresponding phosphate + LPS groups yielded 85, 88, and 91 DEPs, respectively. Common DEPs across the different phosphate + LPS dose groups compared to the LPS model group were also identified (Supplementary Figures S2 and S3).

**Figure 4:**
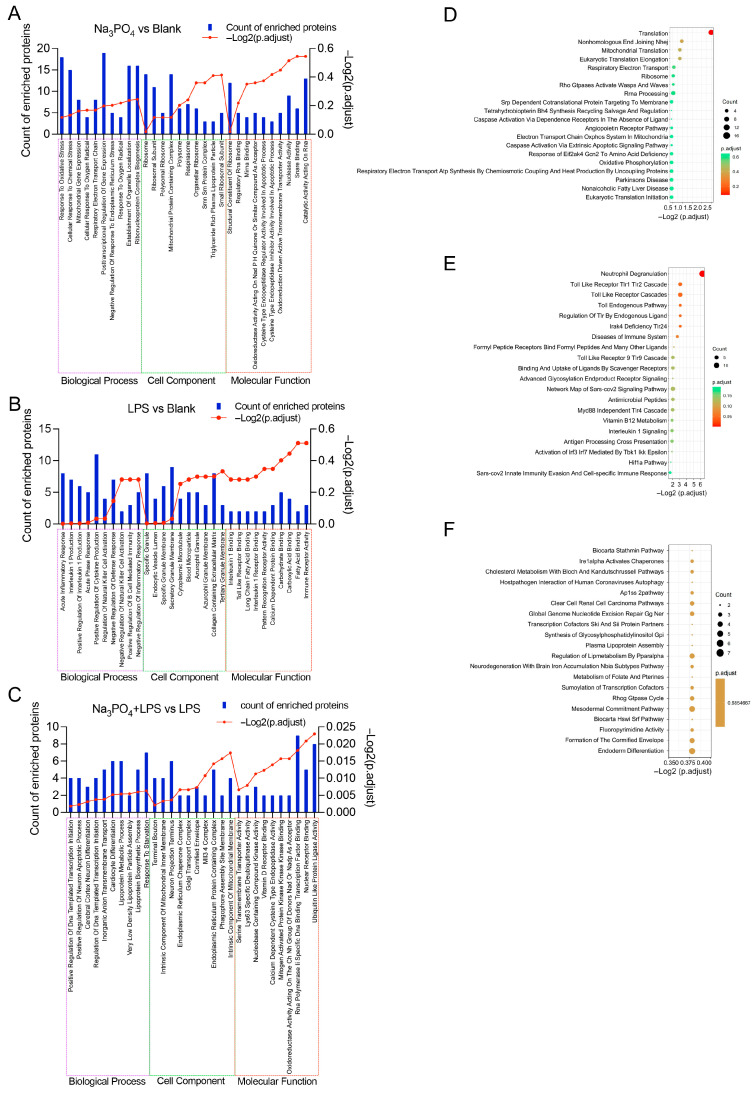
Proteomic analysis of the effects of a high-phosphorus diet on the immune system in mice. (**A**) Top 10 GO functional entries with significant differences between the blank control group and the phosphate group. (**B**) Top 10 GO functional entries ranked by significance between the blank control group and the LPS model group. (**C**) Top 10 GO functional entries ranked by significance in the LPS model group and phosphate–LPS group. (**D**) KEGG pathway enrichment analysis of the blank control group and phosphate group. (**E**) KEGG pathway enrichment analysis of the blank control group and LPS model group. (**F**) KEGG pathway enrichment analysis of LPS model group vs. the phosphate–LPS group.

Gene Ontology (GO) functional annotation analysis suggested potential enrichment of differentially expressed proteins in biological processes related to immune response, cellular stress, and metabolic regulation in the spleen (Figure 4A–C). These pathway associations are based solely on bioinformatic analysis of proteomic data and require functional validation to confirm their biological significance in phosphate overload-induced immune alterations. Kyoto Encyclopedia of Genes and Genomes (KEGG) pathway enrichment analysis indicated that these proteins are primarily involved in signaling pathways including neutrophil degranulation, the ribosome pathway, and DNA damage repair (Figure 4D–F).

Of particular note, within the JAK/STAT signaling pathway, ciliary neurotrophic factor receptor (CNTFR) protein expression was significantly upregulated, while Bcl2l1 protein expression was significantly downregulated (Supplementary Figure S4A). These changes may be associated with immune cell apoptosis and immune organ atrophy. Protein–protein interaction (PPI) network analysis further elucidated the regulatory relationships among the DEPs (Supplementary Figure S4B).

**Figure 5:**
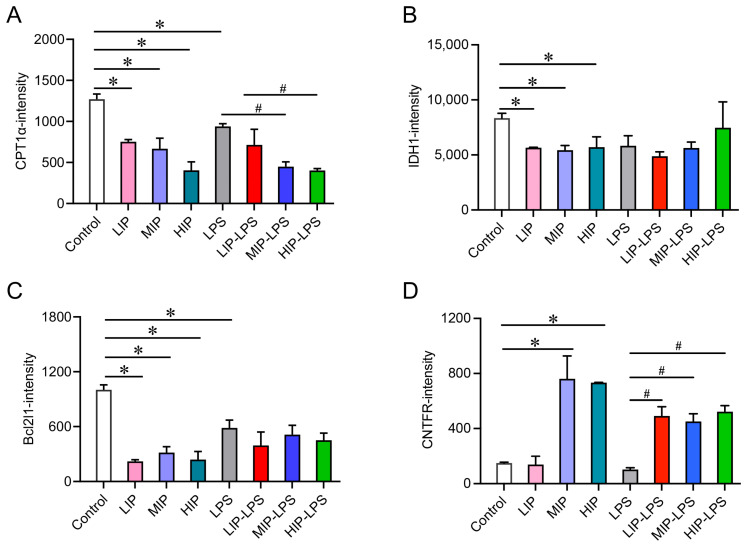
Effects of different phosphate concentrations and LPS stimulation on differentially expressed proteins. (**A**) Changes in CPT1α protein. (**B**) Changes in IDH1 protein. (**C**) Changes in Bcl2l1 protein. (**D**) Changes in CNTFR protein. All data are shown as the mean ± SEM. * *p* < 0.05 vs. controls, ^#^ *p* < 0.05 vs. LPS, determined by two-way ANOVA coupled to Tukey’s HSD post hoc test.

In-depth analysis of key functional proteins revealed that the expression of carnitine palmitoyltransferase 1 alpha (CPT1α) and isocitric dehydrogenase1 (IDH1) was significantly downregulated in the phosphate treatment groups, showing a dose-dependent change (Figure 5A,B). The expression of Bcl2l1 protein was significantly downregulated across all treatment groups (Figure 5C), whereas CNTFR protein expression was significantly upregulated (Figure 5D). The mRNA expression levels corresponded well with those at the protein level (Supplementary Figure S5). These results indicate that a high-phosphate diet alters the splenic protein expression profile, which may be associated with changes in immune regulation, cellular metabolism, and apoptosis-related pathways. This study provides molecular-level evidence for a deeper understanding of the immunoregulatory mechanisms of a high-phosphate diet and offers a scientific basis for assessing the safety of dietary phosphate additives.

## 4. Discussion

This study systematically investigated the comprehensive effects of a high-inorganic-phosphorus diet on the intestinal mucosal barrier, immune environment, and splenic protein expression profiles in mice through integrated in vivo animal models and proteomic analysis. Our preliminary findings suggest that excessive inorganic phosphorus intake may be associated with reduced indices of immune organs such as the thymus and spleen and structural alterations of intestinal villi in mice, decreases goblet cell numbers, and thins the mucus layer, indicating significant alterations in both the intestinal physical structure and immune organ indices. Further analysis of immunoglobulins and cytokines revealed widespread reductions in key immunoglobulins (sIgA, IgA, IgM, IgG) and suppressed IFN-α expression [15]. Secretory immunoglobulin sIgA, serving as the frontline defense of the body’s mucosal system, encapsulates bacteria, viruses, and toxins to prevent pathogen adhesion and penetration of mucosal epithelial cells, thereby avoiding hypersensitivity reactions [16]. The observed reduction in sIgA expression in small intestinal tissues suggests that excessive inorganic phosphate intake may impair the mucosal immune function of the small intestine [17].

Consistent with the study by Sugihara et al. [18], we confirmed the damaging effect of a high-phosphate diet on intestinal barrier function, but there are key differences between the two studies: Sugihara et al. used a dextran sulfate sodium (DSS)-induced severe colitis model and focused on the exacerbating effect of phosphate on pre-existing severe intestinal inflammation; in contrast, our study employed a low-dose lipopolysaccharide (LPS)-induced subclinical inflammation model that more closely mimicked the physiological state of mild intestinal inflammation in humans during daily exposure. Knoepfel et al. [19] further confirmed at the cellular transport mechanism level that high phosphate disrupts intestinal epithelial tight junctions and increases intestinal permeability, which is highly consistent with our observations of intestinal villus atrophy and crypt hyperplasia. In addition, a clinical study by Driman et al. [20] as early as 1998 found that oral sodium phosphate bowel preparation solution causes acute inflammatory reactions and abnormal cell proliferation in the colonic mucosa of healthy individuals, providing direct clinical evidence supporting the potential applicability of our animal experimental results in humans. More importantly, our study is the first to reveal the systemic effects of a high-phosphate diet on immune organs such as the thymus and spleen, and found that high phosphate combined with mild inflammatory stimulation significantly increases intestinal IgE levels, providing the first experimental evidence for the potential association between excessive phosphate intake and food hypersensitivity, an important finding not addressed in previous studies.

LPS can act as an adjuvant in inducing food hypersensitivity reactions. Our results showed that the IgE content in the small intestine of mice was significantly increased after intragastric administration of 200 mg/kg sodium phosphate for 15 days combined with LPS stimulation, demonstrating that high phosphate intake combined with LPS stimulation results in elevated intestinal IgE levels. The observed elevation in intestinal IgE levels raises the exploratory hypothesis that high phosphate intake may increase susceptibility to food hypersensitivity. However, this remains speculative and requires direct validation using allergen exposure models and functional immune assays. No functional assessments were performed in this study to confirm actual immune dysfunction or clinical relevance. As the primary indicator of food hypersensitivity, IgE levels show a significant positive correlation with the degree of intestinal mucosal barrier damage [21]. The elevated IgE observed in this experiment may result from the synergistic damaging effect of sodium phosphate and LPS on the small intestinal mucosa: increased intestinal permeability allows macromolecular allergens from food to enter the bloodstream, stimulating antibody formation and ultimately triggering food hypersensitivity reactions. The significant and complex changes in immunoglobulins (IgA, sIgA, IgE, IgM, IgG) and pro-inflammatory cytokines (IL-1β, IFN-α) in the blood and small intestine of mice caused by the oral administration of inorganic phosphate indicate that excessive inorganic phosphate intake alters the expression profile of immunoglobulins and pro-inflammatory cytokines in the intestinal mucosa.

In terms of immune regulation, our results complement the study by Roberts et al. [15], together confirming the immunomodulatory effects of dietary phosphate. Roberts et al. focused on adaptive immunity and mainly analyzed changes in splenic T cell subsets and cytokines such as IFN-γ and IL-17, while our study focused on mucosal immunity and humoral immunity, systematically detecting changes in the full spectrum of immunoglobulins in serum and intestinal tissues. In their systematic review, Heyer et al. [17], pointed out that high phosphate intake alters the distribution and function of intestinal immune cells and reduces the body’s resistance to pathogens, which is highly consistent with our observation of generally decreased immunoglobulin levels and impaired humoral immune function. Methodologically, Roberts et al. used a dietary supplementation approach to administer high-phosphate diets, which may have dose inaccuracies due to differences in mouse food intake; in contrast, our study used a gradient dose gavage method to precisely control phosphate intake per mouse, eliminating confounding factors from feeding behavior and resulting in more reliable results.

Notably, although our study did not directly measure gut microbiota, the observed intestinal barrier damage, mucus layer thinning, and immunoglobulin profile changes are highly consistent with host immune phenotypes induced by gut microbiota dysbiosis. Oda et al. [22] reported that a high-phosphate diet significantly disrupts the composition and diversity of gut microbiota in mice but did not further explore the impact of dysbiosis on host immunity. Favero et al. [23,24] found a vicious cycle between hyperphosphatemia, gut microbiota dysbiosis, intestinal barrier dysfunction, and systemic chronic inflammation in patients with chronic kidney disease. These findings collectively suggest that the “phosphate overload–gut microbiota dysbiosis–host immune injury” axis may be one of the core mechanisms underlying the adverse effects of excessive phosphate intake, pointing to an important direction for future research.

At the molecular mechanism level, proteomic results revealed alterations in multiple key proteins and signaling pathways in mouse spleen tissues. CNTFR is a neurotrophic factor receptor, and its signaling pathway is critical for the survival, development, and repair of motor neurons. In certain disease states (e.g., amyotrophic lateral sclerosis [ALS]), the upregulation of CNTFR is often regarded as a compensatory response [25,26]. Bcl2l1 is a key anti-apoptotic protein in the Bcl-2 family, playing a central role in the mitochondrial apoptosis pathway. Its downregulation significantly impairs the anti-apoptotic capacity of cells, making them more susceptible to programmed cell death under stress [27]. Its downregulation suggests that the ability of cells to utilize fatty acids for energy production may be reduced, which may lead to energy metabolism disorders and lipid accumulation. IDH1 catalyzes the oxidative decarboxylation of isocitrate to produce α-ketoglutarate (α-KG) in the cytoplasm while also generating NADPH. Its downregulation results in a decrease in the cellular antioxidant NADPH, making cells more vulnerable to oxidative stress damage; it also reduces α-KG levels, disrupting various cellular metabolic and epigenetic regulatory processes [28,29].

Phosphorus overload affected various pathways, including oxidative stress, mitochondria gene expression, respiratory electron transport chain, post-transcriptional regulation of gene expression, endoplasmic reticulum stress, and the biosynthesis of ribonucleoprotein complexes [30]. Hsu et al. [31] confirmed in cardiac research that a high-phosphate diet causes atrial remodeling by activating the STAT3/NF-κB signaling pathway; Fujimura et al. [32] found in renal cells that high phosphate exposure induces mitochondrial dysfunction and increased reactive oxygen species production; Hetz et al. [25] found through quantitative proteomic analysis that high phosphate widely disrupts intracellular signal transduction pathways. Our study is the first to find that high phosphate can simultaneously affect the JAK/STAT immune regulatory pathway and mitochondrial metabolic pathway in spleen tissue, providing a new molecular mechanism explanation for the systemic immunotoxicity of phosphate overload.

The observed expression changes of CNTFR, Bcl2l1, CPT1α, and IDH1 are consistent with molecular alterations reported in some ALS animal models. Nevertheless, this only represents an observation of molecular resemblance at the splenic level. No evidence is provided in this study to support a causal link between dietary phosphate intake and neurodegenerative disorders, and this potential association remains an untested hypothesis requiring independent verification.

Based on the experimental data presented in this study, we propose a preliminary, evidence-based mechanistic model: high inorganic phosphate intake first induces structural damage to the intestinal mucosa, including villus atrophy, crypt hyperplasia, and reduced goblet cell numbers. This impairs the intestinal physical barrier function, leading to increased intestinal permeability. Luminal antigens and bacterial products then translocate into the systemic circulation and activate immune cells in the spleen and other secondary lymphoid organs. This immune activation is associated with altered expression of immunoglobulins and cytokines. At the molecular level, we observed changes in key proteins in the spleen: upregulation of CNTFR and downregulation of Bcl2l1 in the JAK/STAT signaling pathway, as well as downregulation of metabolic regulators CPT1α and IDH1. These molecular changes may further contribute to immune dysregulation by affecting immune cell survival, proliferation, and energy metabolism. This model is based on the integration of our experimental findings. However, the causal relationships between these sequential events have not been directly demonstrated in this study. Future studies using tissue-specific gene manipulation and adoptive transfer experiments are needed to confirm this mechanistic framework. It should be emphasized that this study investigated the acute effects of 15 days of high-inorganic-phosphate exposure in mice. In contrast, human exposure to excessive dietary phosphate is typically chronic, occurring over years or decades. The acute effects observed in this study may not directly translate to the long-term effects of chronic low-level exposure in humans. Further long-term animal studies and epidemiological investigations are needed to establish the relationship between chronic phosphate intake and human health outcomes.

This exploratory study provides preliminary descriptive data on the effects of high-inorganic-phosphate diets on intestinal structure and immune-related molecular profiles in mice. It provides preliminary molecular evidence that the JAK/STAT signaling pathway and cellular metabolic reprogramming may play potential roles in these effects. These findings provide preliminary experimental evidence that may inform future safety evaluations of inorganic phosphate food additives and offer new research directions for the development of prevention and rehabilitation strategies for phosphorus overload-related chronic diseases.

The strength of our conclusions is appropriately constrained by the limitations of the study design. All findings need to be validated through functional verification experiments in larger-scale and long-term studies before any definitive statements can be made regarding the impacts of dietary phosphorus on human health.

In summary, the main contributions of our study to the existing literature are reflected in three aspects: (1) It is the first to systematically reveal the dose-dependent effects of inorganic phosphate on intestinal mucosal barrier and systemic immune homeostasis in mice, clarifying the degree of damage at different exposure levels; (2) It provides the first experimental evidence that a high-phosphate diet combined with mild inflammatory stimulation increases intestinal IgE levels, proposing a new hypothesis that phosphate food additives may increase the risk of food hypersensitivity; and (3) It identifies novel molecular targets related to the JAK/STAT pathway and mitochondrial metabolism through splenic proteomics, laying the foundation for further elucidating the immunotoxic mechanisms of phosphate overload.

## 5. Conclusions

This study employed a gradient inorganic phosphate (Pi) gavage model combined with low-dose LPS-induced subclinical inflammation in mice to investigate the effects of excessive dietary Pi on intestinal mucosal barrier function and systemic immune homeostasis. High Pi intake dose-dependently reduced immune organ and kidney indices, damaged the intestinal mucosal barrier, and altered immunoglobulin and cytokine expression profiles. Notably, high Pi combined with LPS significantly elevated intestinal IgE levels, supporting the hypothesis that excessive Pi food additives may increase food hypersensitivity susceptibility. Splenic proteomics further identified key proteins in the JAK-STAT and mitochondrial metabolic pathways, revealing potential molecular mechanisms. These findings expand our understanding of Pi’s health risks and provide experimental basis for its safety evaluation.

## Data Availability

The data reported in this paper have been deposited in the OMIX, China National Center for Bioinformation/Beijing Institute of Genomics, Chinese Academy of Sciences (https://ngdc.cncb.ac.cn/omix: accession no.OMIX014618, accessed on 25 January 2026) [33,34].

## Supplementary Materials

The following supporting information can be downloaded at: https://www.mdpi.com/article/10.3390/nu18101590/s1. Figure S1. Analysis of Proteomics Data Quality Control Results; Figure S2. Analysis of Differentially Expressed Proteins; Figure S3. Analysis of Differentially Expressed Proteins; Figure S4. JAK-STAT pathway and DEP interaction network analysis; Figure S5. Effects of different phosphate concentrations and LPS stimulation on gene expression; Table S1. The primers used in the RT-qPCR. Limitations: This study has several key limitations, including small sample size, acute exposure model, lack of functional immune assays, and tissue-specific proteomic analysis. A comprehensive list of limitations is provided in the Supplementary Materials.
