# Supplementary material for: Effects of High-Inorganic-Phosphorus Diet on Intestinal Mucosal Injury and Immune Alteration in Mice
Source: Nutrients. 2026 May 16;18(10):1590. doi: 10.3390/nu18101590 (PMC13209458; doi:10.3390/nu18101590)
Supplement: Supplementary file 1 [file nutrients-18-01590-s001.zip › nutrients-4247285-supplementary.pdf]

Supplementary Information

Effects of High-Inorganic-Phosphorus Diet on Intestinal Mucosal Injury and Immune Alterations in Mice

Zongchao Sun[1#], Shiya Huang[1#], Yuxin Zhao[2], Yunhan Luan[1], Yinuo Wang[1], Runzhe Wang[1], Weiwei Wu[2], Danli Huang[2], Jiankang Liu[1], Yinghui Zhang[1*]
1School of Life Sciences and Health, University of Health and Rehabilitation Sciences, Qingdao, 266113, China; sunzongchao@uhrs.edu.cn; huangshiya@uhrs.edu.cn
2School of Food Science and Engineering, Foshan University, Foshan, 528225, China; zhaoyx202407@163.com
*Correspondence: zhangyinghui@uhrs.edu.cn; Tel.: 86-532-57787388;
[#]Those authors made equal contributions to the study.

Contents:

1. Method
2. Supplementary Figures S1-S5.
3. Limitations
4. Supplementary Table S1.

Method

1. Preparation of Paraffin Sections

After obtaining small intestinal tissue from experimental mice, the intestinal con-tents were thoroughly rinsed with PBS solution or normal saline. The small intestinal tissue was fixed in Carnoy's fixative for at least 4 hours. The fixed intestinal tissue was then removed and cut into segments approximately 1 cm in length. The intestinal segments were sequentially dehydrated in ethanol solution containers. The dehydration ethanol concentration was as follows: first immersed in 85% ethanol solution for 1 hour, then in 95% ethanol solution for 1 hour (repeated twice), and finally in anhydrous ethanol solution for 1 hour (repeated twice). The dehydrated intestinal tissue was then transparentized. After completion of the transparentization process, paraffin embed-ding was performed. The transparentization and embedding steps were as follows: the tissue was immersed in two xylene solution containers for 30 minutes each, followed by immersion in three molten paraffin containers for 30 minutes, 25 minutes, and 20 minutes, respectively. The paraffin-embedded small intestinal tissue was placed in the embedding container. During the rapid pouring of molten paraffin, efforts were made to minimize bubble formation. The paraffin was then placed in an ice bath to accelerate solidification. Once the paraffin was fully solidified and hardened, the tissue embed-ding was complete. The processed paraffin blocks were sectioned. The paraffin blocks were placed in a tissue slicer for sectioning. The cut paraffin sections were transferred to a constant-temperature water bath for slow unfolding. Unfolded tissue sections were removed from the water bath using a slide, ensuring that the small intestinal tissue sections were completely adhered to the slide.

2. Preparation of Intestinal HE-Stained Sections

Preparation method for HE-stained tissue sections: Paraffin sections were immersed twice in xylene solution (20 minutes each time) and then twice in anhydrous ethanol (5 minutes each time). Subsequently, they were immersed in 75% ethanol for 5 minutes. Finally, the sections were rinsed with tap water. After dewaxing, the sections were stained with hematoxylin for approximately 4 minutes. Following hematoxylin staining, the sections were rinsed with tap water and then decolorized with a decolorizing solution. After decolorization, the sections were rinsed again with running water. The sections were then re-stained with a re-staining solution and rinsed with running water. For eosin staining of hematoxylin-stained sections: The

sections were first dehydrated with 85% ethanol for 5 minutes, then with 95% ethanol for 5 minutes, followed by 5 minutes of eosin staining. After eosin staining, the sections were dehydrated by soaking in anhydrous ethanol three times (5 minutes each time). Subsequently, they were immersed twice in xylene solution (5 minutes each time) until the sections became transparent and decolorized. After completing the above steps, the sections were sealed. After sealing the small intestinal tissue sections with HE staining, the staining changes were observed under a forward optical microscope. Randomly selected 3-5 mouse intestinal villi were photographed to measure villus length and crypt depth, and the ratio of villus length to crypt depth was calculated.

3. Preparation of AB-PAS-Stained Intestinal Sections

Mouse small intestinal paraffin sections were routinely deparaffinized to water, then placed in 1% Alcian Blue acetic acid staining solution for 15 min at room temperature, followed by thorough rinsing with running tap water until the rinse water was colorless. Subsequent to Alcian Blue staining, the sections were transferred to 1% periodic acid solution for 15 min incubation at room temperature, quickly rinsed once with tap water and then washed with distilled water to remove residual periodic acid. After that, the sections were incubated in Schiff's staining solution for 30 min at room temperature in the dark, rinsed with running tap water, immersed in hematoxylin staining solution for 2 min counterstaining, and then rinsed with distilled water. Finally, the sections were dehydrated, cleared and mounted using the same routine method as HE-stained sections; after preparation, the morphological changes of small intestinal tissues were observed under an inverted light microscope, with intestinal lumen villi photographed and goblet cells on the villi counted and analyzed.

4. ELISA Assay for Detecting Serum sIgA and IgE Levels in Mice

Prior to the assay, standard solutions designated as S1 to S8 (unit: ng/ml) were prepared, with S8 serving as the sample diluent and the concentrations being 10, 5, 2.5, 1.25, 0.63, 0.31, 0.16, and 0 ng/ml, respectively; the reagents and materials used included the aforementioned standard solutions, biotin-labeled secretory immunoglobulin antibody working solution, horseradish peroxidase (HRP)-labeled streptavidin working solution, wash buffer, chromogenic reagent, stop solution, 96-well microplate, and a pre-warmed microplate reader for absorbance measurement. For the experimental procedures, standard wells (with duplicate wells to ensure reproducibility) and sample wells were first set up on the 96-well microplate, followed by

adding 100 μl of standard working solution or 100 μl of each serum sample to the corresponding wells; after loading, the microplate was sealed and incubated at 37°C for 90 minutes, and the liquid in each well was discarded thereafter with the microplate gently tapped on absorbent paper to remove residual liquid. Subsequently, 100 μl of biotin-labeled secretory immunoglobulin antibody working solution was added to each reaction well, the microplate was resealed and incubated at 37°C for 1 hour, and the first washing step was performed by adding 350 μl of pre-prepared wash buffer to each well (standing for 2 minutes before discarding), repeating this step 4 times with the microplate tapped dry to remove excess wash buffer prior to each subsequent wash. After washing, 100 μl of HRP-labeled streptavidin working solution was added to each well, the microplate was sealed and incubated at 37°C for 30 minutes, followed by a second washing step where 300 μl of wash buffer was added to each reaction well (standing for 30 seconds before discarding), repeating this procedure 4 times and thoroughly removing residual wash buffer by vortexing after the final wash. Under dark conditions, 90 μl of pre-prepared chromogenic reagent was then added to each well, the microplate was sealed and incubated at 37°C in the dark for approximately 15 minutes to facilitate color development, and immediately after color development, 50 μl of stop solution was added to each well to terminate the reaction.

5. ELISA assay for detecting IFN-α and IL-1β levels, as well as IgE, IgG, IgM, and IgA expression in mouse small intestinal tissue

Tissue samples were homogenized into a homogeneous slurry, followed by ultrasonic disruption for complete lysis; after lysis, the samples were centrifuged at 5000 × g for 5–10 min, and the supernatant was collected and stored appropriately for subsequent assay, while standard stock solutions were serially diluted to generate a series of gradient-concentration working standards that were aliquoted and preserved under optimal conditions until use; for the assay, a 96-well reaction plate was prepared with blank wells and sample wells arranged according to the experimental design, 100 μL of each sample supernatant or gradient standard was added to the corresponding wells, subsequently 50 μL of biotinylated antibody working solution was added to each well, and the plate was sealed and incubated at 20–25 °C with shaking at 300 rpm on a microplate shaker for 120 min; after the first incubation, the plate was subjected to washing by dispensing 300 μL of wash buffer into each well, incubating for 10 s, then completely removing the liquid

by gentle shaking, a procedure repeated 5 times, followed by adding 100 μL of enzyme conjugate working solution to all wells except the blank wells, resealing the plate and incubating at 20–25 °C with shaking at 300 rpm for 60 min; a second washing step was then performed by adding 300 μL of wash buffer to each well, incubating for 10 s, thoroughly removing residual wash buffer by centrifugation, and repeating this step 5 times to eliminate non-specific binding; under dark conditions, 100 μL of chromogenic substrate was added to each reaction well, the plate was sealed immediately after reagent addition and incubated at 20–25 °C in the dark for 15 min to allow color development, and upon completion of color development, 100 μL of stop solution was promptly added to each well to terminate the reaction.

6. Immunofluorescence Detection of IgA Cell Expression in the Intestinal Tract of Experimental Mice

An appropriate volume of environmentally friendly dewaxing solution and anhydrous ethanol were separately prepared in different containers. Paraffin sections were first immersed in three sequential containers of environmentally friendly dewaxing solution for 10 min each, then transferred to three sequential containers of anhydrous ethanol for 5 min each. After this treatment, the sections were rinsed with distilled water prior to antigen retrieval. Antigen retrieval was performed under the following conditions: microwave irradiation at medium power for 8 min in citric acid buffer (pH 6.0), followed by standing for 8 min, and subsequent microwave irradiation at low-medium power for 7 min. After fixation, the sections were placed in phosphate-buffered saline (PBS, pH 7.4) and washed on a decolorizing shaker for 5 min per wash, with this washing step repeated three times. For serum blocking, a histochemical pen was used to draw circles around the treated tissue, followed by dropwise addition of bovine serum albumin (BSA) for blocking at room temperature for 30 min. Subsequent to serum blocking, primary antibody was pipetted onto the tissue sections, and the sections were incubated overnight at 4 °C. The incubated sections were then washed in PBS (pH 7.4) on a decolorizing shaker three times, 5 min per wash. Next, the corresponding secondary antibody was added to the sections, which were then incubated at room temperature in the dark for 50 min. After secondary antibody incubation, the sections were washed in PBS (pH 7.4) three times for 5 min each on the decolorizing shaker. Nuclear staining was performed using 4',6-diamidino-2-phenylindole (DAPI) reagent: DAPI solution was added dropwise to the sections, followed by incubation at room temperature in the

dark for 10 min. The stained sections were then washed again in PBS (pH 7.4) three times, 5 min per wash. Subsequently, pre-prepared autofluorescence quencher was added to the sections and allowed to stand for 5 min. Finally, the sections were rinsed with running water, and the processed sections were mounted using anti-autofluorescence mounting medium. Following these procedures, image acquisition was conducted.

Supplementary Figure

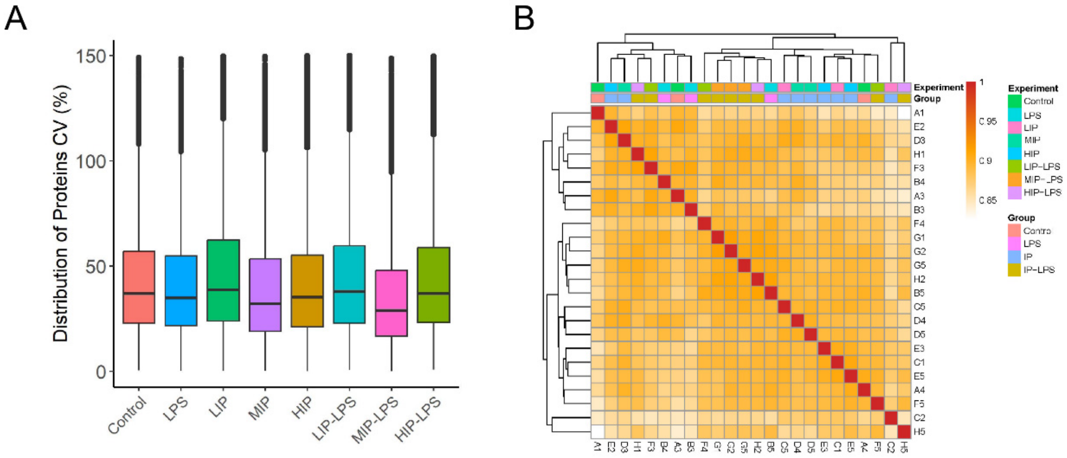

Supplementary Figure S1. Analysis of Proteomics Data Quality Control Results. A) Coefficients of variation (CV) of protein quantification across samples within each experimental group. Low CV values indicate high reproducibility and consistency among biological replicates. B)Correlation matrix of protein quantification between samples. Pearson correlation coefficients (r) are shown; values close to 1 indicate strong correlations, demonstrating reliable data quality for downstream differential expression analysis.

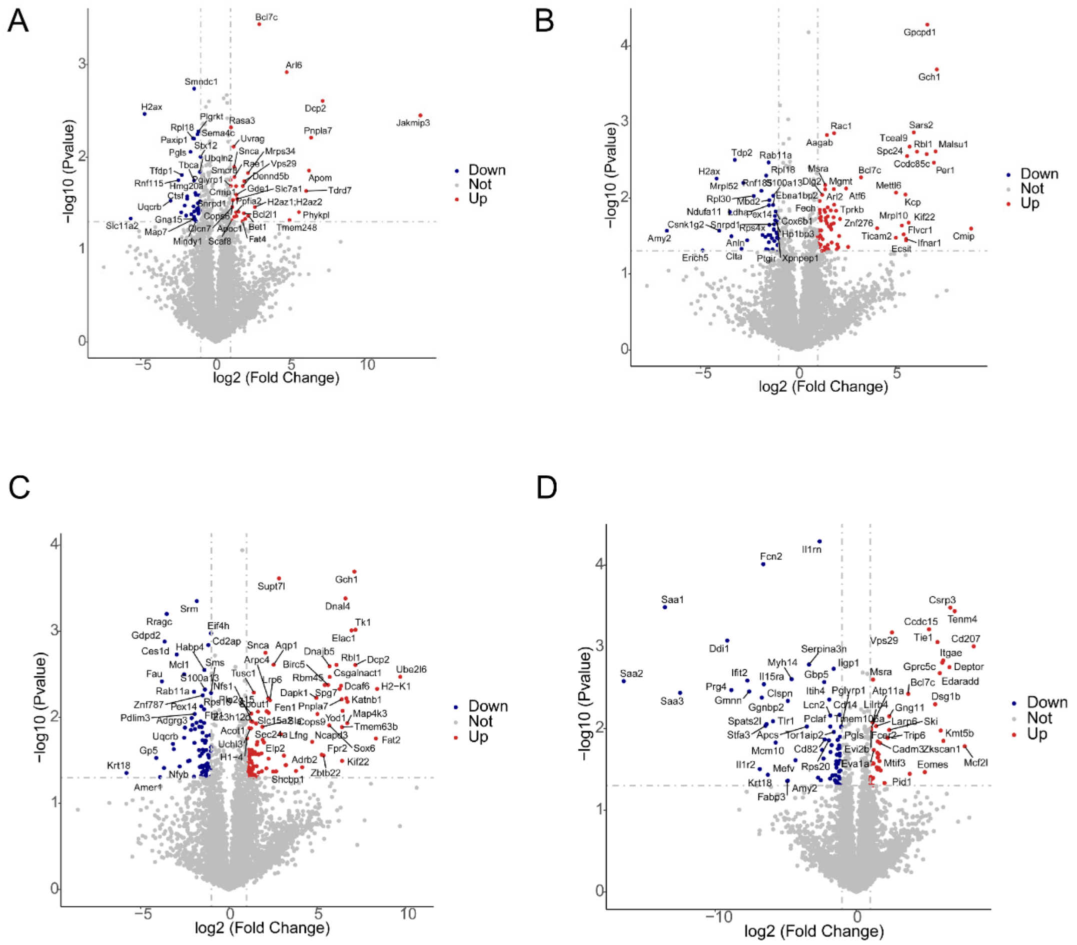

Supplementary Figure S2. Analysis of Differentially Expressed Proteins. A) Blank control vs. LIP B) Blank control vs. MIP C) Blank control vs. HIP D) Blank control vs. LPS

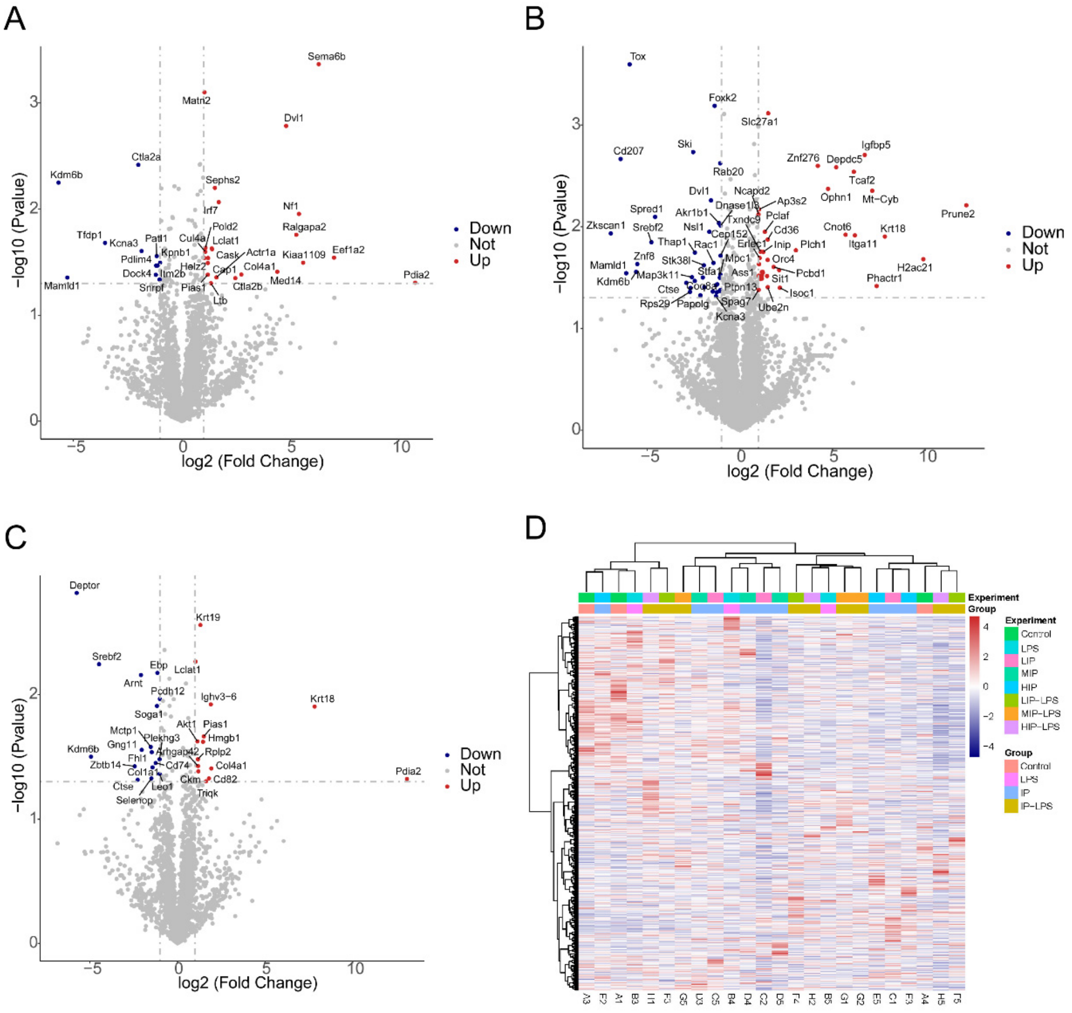

Supplementary Figure S3. Analysis of Differentially Expressed Proteins. Volcano Plot of Differentially Expressed Proteins and Hierarchical Clustering Analysis of Differentially Expressed Proteins. A) LPS model vs. LIP+LPS **B)** LPS model vs. MIP+LPS **C)** LPS model vs. HIP+LPS **D)** The heatmap shows the expression patterns of representative differentially expressed proteins (rows) under the indicated treatment groups. The color scale represents the log_2 (fold change) relative to the control, with blue indicating downregulation and red indicating upregulation.

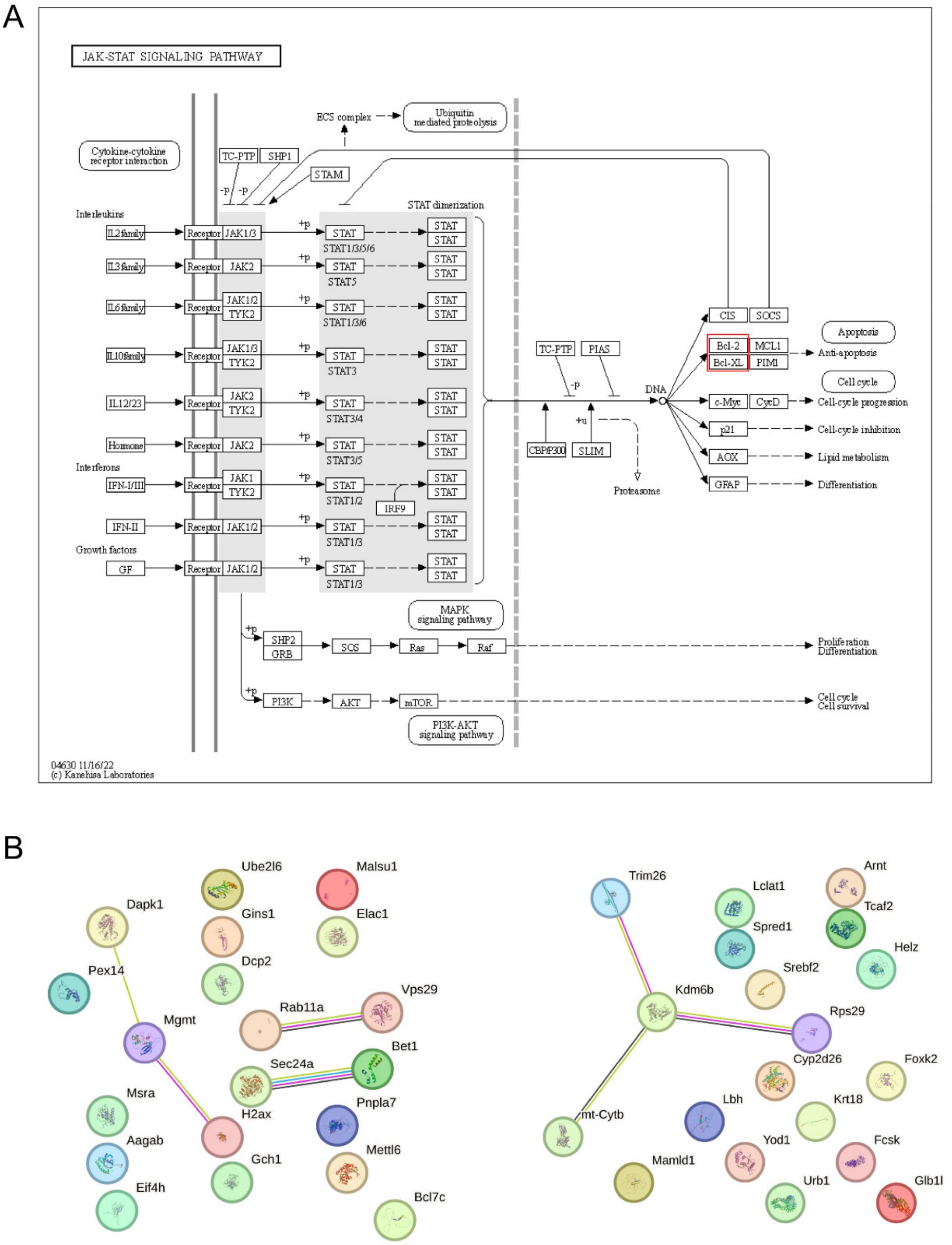

Supplementary Figure S4. JAK-STAT pathway and DEP interaction network analysis. **A)** Schematic representation of the JAK-STAT signaling pathway. Within this pathway, ciliary neurotrophic factor receptor (CNTFR) protein expression was significantly up-regulated, while Bcl2l1 protein expression was significantly down-regulated in response to high inorganic phosphate treatment, as identified by proteomic analysis. These changes may be associated with immune cell apoptosis and immune organ atrophy. **B)** Protein-protein interaction (PPI) network analysis elucidating the regulatory relationships among the differentially expressed proteins (DEPs). Nodes represent proteins, and edges indicate known or predicted interactions (e.g., from the STRING database). The network includes key DEPs

from this study, highlighting potential hub proteins and clusters involved in immune regulation, mitochondrial metabolism, and apoptosis.

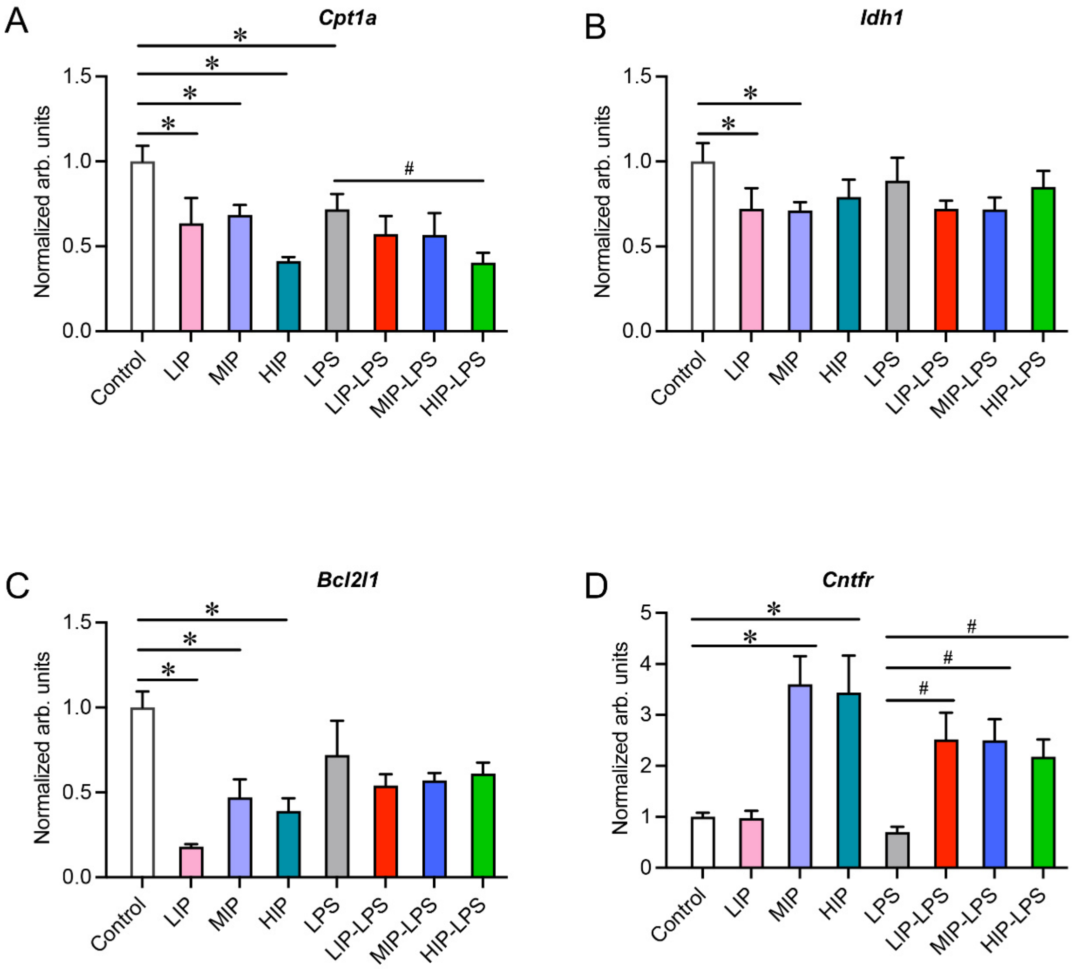

Supplementary Figure S5. Effects of different phosphate concentrations and LPS stimulation on gene expression. **A)** *Cpt1a* mRNA expression. **B)** *Idh1* mRNA expression. **C)** *Bcl2l1* mRNA expression. **D**) *Cntfr* mRNA expression.

Limitations

This study has several limitations that should be acknowledged. First, the sample size of five mice per group is relatively small. Although this sample size is standard for preliminary exploratory studies and is sufficient to detect the large effect sizes observed in the key findings of this work, it limits the statistical power to identify moderate or small effect sizes and increases the risk of false-positive errors in multiple comparisons across eight experimental groups with multiple endpoints, including histological, immunological, and proteomic outcomes. Therefore, all conclusions drawn in this study should be regarded as preliminary. Further studies with larger sample sizes are required to validate and extend our findings. Second, only female BALB/c mice were used to eliminate the confounding effects of the estrous cycle on immune function, which limits the generalizability of the findings to male animals, other mouse strains, or humans. Third, the 15-day exposure period only allowed for an investigation of the acute effects of high inorganic phosphate intake, and long-term chronic exposure studies are needed to evaluate progressive organ damage. The acute effects observed in this study may not directly translate to the long-term effects of chronic low-level exposure in humans. Fourth, while we have validated the expression changes of four key differentially expressed proteins (CPT1α, IDH1, Bcl2l1, and CNTFR) at the mRNA level using qPCR, the expression of other differentially expressed proteins has not been independently verified. Furthermore, this study only confirmed the correlation between the expression changes of these molecules and phosphate overload-induced phenotypes; their causal roles and specific molecular mechanisms in intestinal mucosal injury and immune dysregulation require further functional validation through in vitro and in vivo gene manipulation experiments. Fifth, the role of the gut microbiota was not directly investigated, although its potential contribution has been discussed. Sixth, this study only measured the levels of immunoglobulins and cytokines, and no functional immune assays (such as lymphocyte proliferation, phagocytosis, or cytotoxicity assays) were performed. Therefore, we cannot conclude that the observed molecular changes translate to actual immune dysfunction. Additionally, no allergen exposure models were used, so the potential link between phosphate intake and food hypersensitivity remains a preliminary hypothesis with no direct experimental support. Future studies should include functional immune assessments and allergen challenge experiments to validate these findings. Seventh, proteomic analysis was performed exclusively on spleen tissue. Therefore, these findings cannot be generalized to other immune organs or

systemic immune responses. The identified protein expression changes and pathway associations are preliminary and limited to the splenic context, requiring validation in additional tissues and functional studies to establish their broader biological relevance. Eighth, the extrapolation of animal dose-response data to humans has inherent limitations. This study used acute oral gavage of pure trisodium phosphate, which does not fully replicate the complexity of human dietary exposure, including differences in food matrix effects, bioavailability, and chronic intake patterns. Therefore, all statements regarding the human relevance of our findings should be considered preliminary and hypothetical. Finally, only organ indices were measured for immune and metabolic organs, and histological and functional validation are required to confirm actual organ damage. These limitations will be systematically ad-dressed in our future research.

Supplementary Table S1 The primers used in the RT-qPCR.

Gene	*Forward (5′→3′)*	*Reverse (5′→3′)*
Cpt1a	*GTGTTGGAGGTGACAGACTT*	*CACTTTCTCTTTCCACAAGG*
Idh1	*CGGTCATGTTCCTACACGGC*	*GACATTCCCGGCATTGTGAT*
Bcl2l1	*GGTGAGTCGGATCGCAGCTTG*	*CTCTCGGCTGCTGCATTGTTC*
Cntfr	*CCAACAGGAAGGAAGGAGTGA*	*CTGGGCTTATCGCCATCTCTT*
Hprt1	*GGACTTGAATCATGTTTGTG*	*CAGATGTTTCCAAACTCAAC*
